# Supplementary material for: Reciprocal regulation of RIG-I and XRCC4 connects DNA repair with RIG-I immune signaling
Source: Nat Commun. 2021 Apr 12;12:2187. doi: 10.1038/s41467-021-22484-7 (PMC8041803; doi:10.1038/s41467-021-22484-7)
Supplement: Supplementary file 1 — Supplementary Information [file 41467_2021_22484_MOESM1_ESM.pdf]

Reciprocal regulation of RIG-I and XRCC4 connects DNA repair with RIG-I immune signaling

Supplementary Information (Supplementary Figures 1-7 and Table 1)

Guo et al.

Supplementary Figure 1

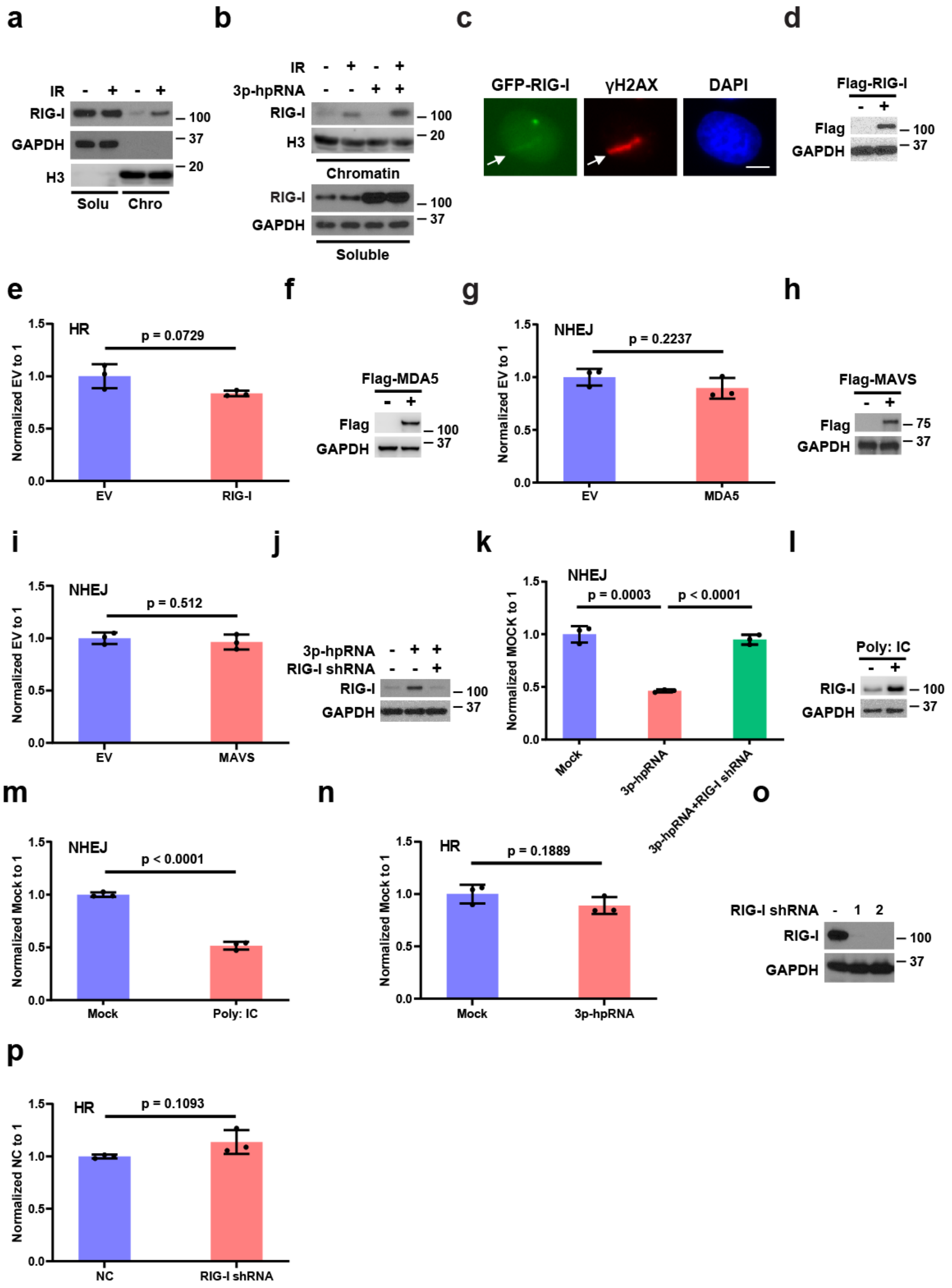

Supplementary Figure 1

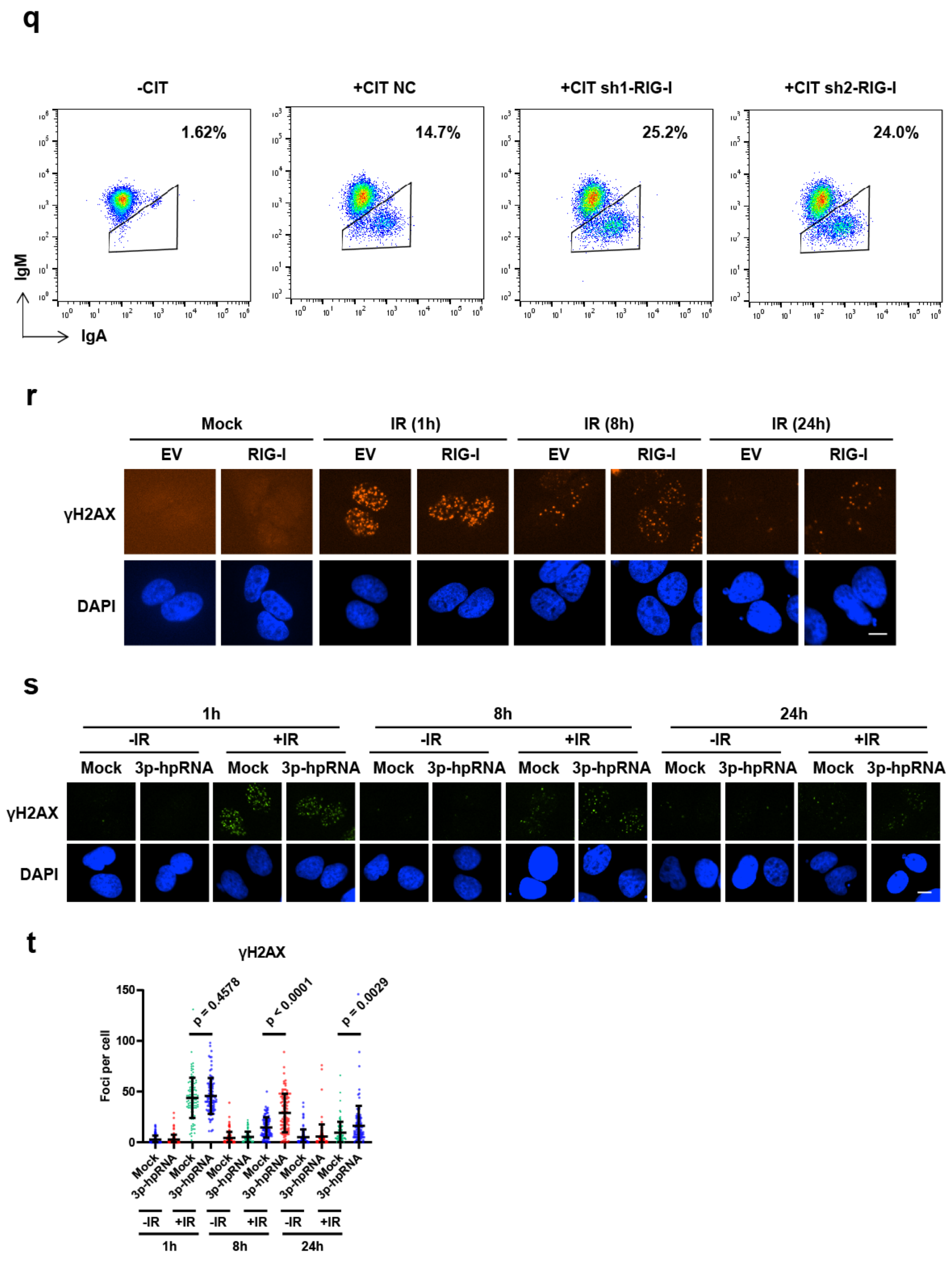

Supplementary Fig. 1: RIG-I is recruited to DNA DSBs and suppresses non-homologous end joining. **a** MDA-MB-231 cells were treated with irradiation (IR, 10 Gy, 2 h). RIG-I protein levels in the soluble (Solu) and chromatin (Chro) fractions were examined by Western blot. **b** HEK293T cells were transfected with 3p-hpRNA (0.5 μg/ml, 12 h), and then treated with IR (10

Gy, 2 h). RIG-I protein levels in the soluble and chromatin fractions were examined by Western blot. **c** GFP-RIG-I (green) localizes to laser-induced DNA damage sites (red, γH2AX) following micro-irradiation. Scale bar, 10 μm. **d-e** Control (EV) and RIG-I overexpressing (RIG-I) HEK293T cells **(d)**, were transfected with HR reporter, and then cells were harvested for HR assay **(e)**. Data are presented as mean values ± SEM from three independent experiments. *P*-values are determined by unpaired two-sided t-test. **f-i** The NHEJ-mediated DSB repair efficiency of MDA5 (**f, g**) or MAVS (**h, i**) overexpressing HEK293T cells, was analyzed using NHEJ reporter. Data are presented as mean values ± SEM from three independent experiments. *P*-values are determined by unpaired two-sided t-test. **j-k** HEK293T cells were transfected with 3p-hpRNA (0.5 μg/ml, 12 h), and then infected with lentiviruses expressing RIG-I shRNA (**j**). The NHEJ-mediated DSB repair efficiency was analyzed using NHEJ reporter (**k**). Data are presented as mean values ± SEM from three independent experiments. *P*-values are determined by unpaired two-sided t-test. **l-m** The NHEJ-mediated DSB repair efficiency of HEK293T cells transfected with Poly: IC (0.5 μg/ml, 12 h) (**l**), was analyzed using NHEJ reporter (**m**). Data are presented as mean values ± SEM from three independent experiments. *P*-values are determined by unpaired two-sided t-test. **n** The HR-mediated DSB repair efficiency of HEK293T cells transfected with 3p-hpRNA (0.5 μg/ml, 12 h), was analyzed using HR reporter. Data are presented as mean values ± SEM from three independent experiments. *P*-values are determined by unpaired two-sided t-test. **o-p** The HR-mediated DSB repair efficiency of control and RIG-I knockdown HEK293T cells (**o**), was analyzed using HR reporter (**p**). Data are presented as mean values ± SEM from three independent experiments. *P*-values are determined by unpaired two-sided t-test. **q** Representative pictures of CSR efficiency in control and RIG-I knockdown CH12F3 cells. CSR efficiency was assessed using flow cytometry analysis of IgM and IgA expression following CIT (TGF-β1, IL-4, and CD40 ligand) treatment. Numbers indicate the percentage of IgA^+ cells. **r** Representative pictures of γH2AX foci in control and RIG-I overexpressing U2OS cells treated with IR (2 Gy) as indicated. Scale bar, 10 μm. **s-t** Representative pictures (**s**) and quantification (**t**) of γH2AX foci in control and 3p-hpRNA treated U2OS cells with or without IR (2 Gy) treatment. Data are representative of three independent experiments. Each dot represents a single cell, and 100 cells were counted in each group for this experiment. Error bars represent ± SEM from this experiment. *P*-values are determined by unpaired two-sided t-test. Scale bar, 10 μm.

Supplementary Figure 2

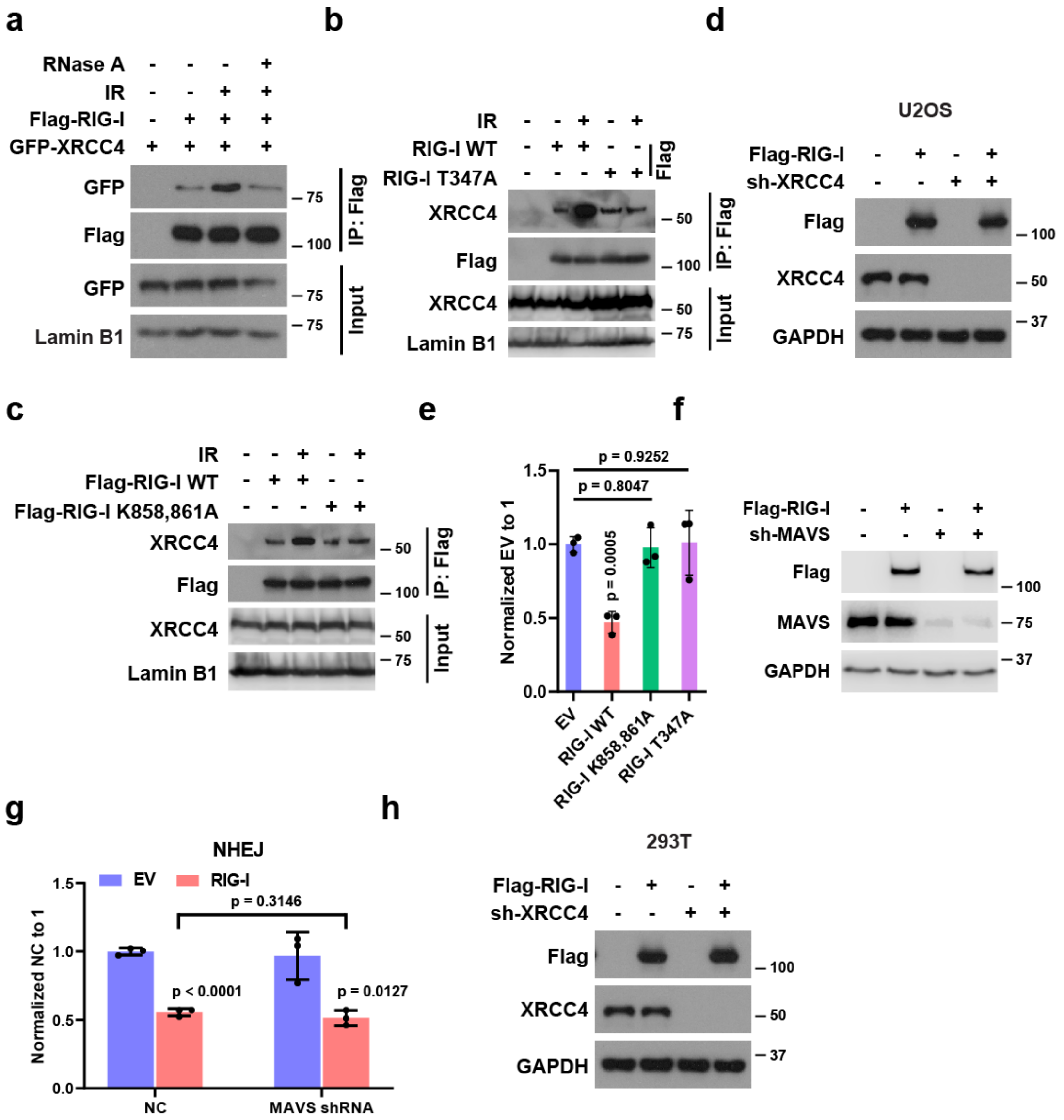

Supplementary Fig. 2: XRCC4 is required for the recruitment of RIG-I to DSB sites. **a** HEK293T cells were transfected with Flag-RIG-I and GFP-XRCC4, and then treated with IR (10 Gy, 2 h). The cells were lysed, and nuclear fractions were immunoprecipitated with anti-Flag agarose beads. The beads were treated with RNase A, boiled and blotted with indicated antibodies. **b-c** HEK293T cells were transfected with Flag-RIG-I T347A **(b)** or Flag-RIG-I K858, 861A **(c)**, and then treated with IR (10 Gy, 2 h). The cells were lysed, and nuclear fractions were immunoprecipitated with anti-Flag agarose beads. The beads were boiled and blotted with indicated antibodies. **d** Western blot analysis of RIG-I and XRCC4 protein levels in control and XRCC4 knockdown U2OS cells overexpressing RIG-I. **e** HEK293T cells overexpressing WT or RIG-I mutants (T347A; K858, 861A), were transfected with NHEJ reporter, and then cells were harvested for NHEJ assay. Data are presented as mean values ± SEM from three independent

experiments. *P*-values are determined by unpaired two-sided t-test. **f-g** Control and MAVS knockdown HEK293T cells overexpressing RIG-I **(f)**, were transfected with NHEJ reporter, and then cells were harvested for NHEJ assay **(g)**. Data are presented as mean values ± SEM from three independent experiments. *P*-values are determined by unpaired two-sided t-test. **h** RIG-I and XRCC4 protein levels in control and XRCC4 knockdown HEK293T cells overexpressing RIG-I, were detected by Western blot.

Supplementary Figure 3

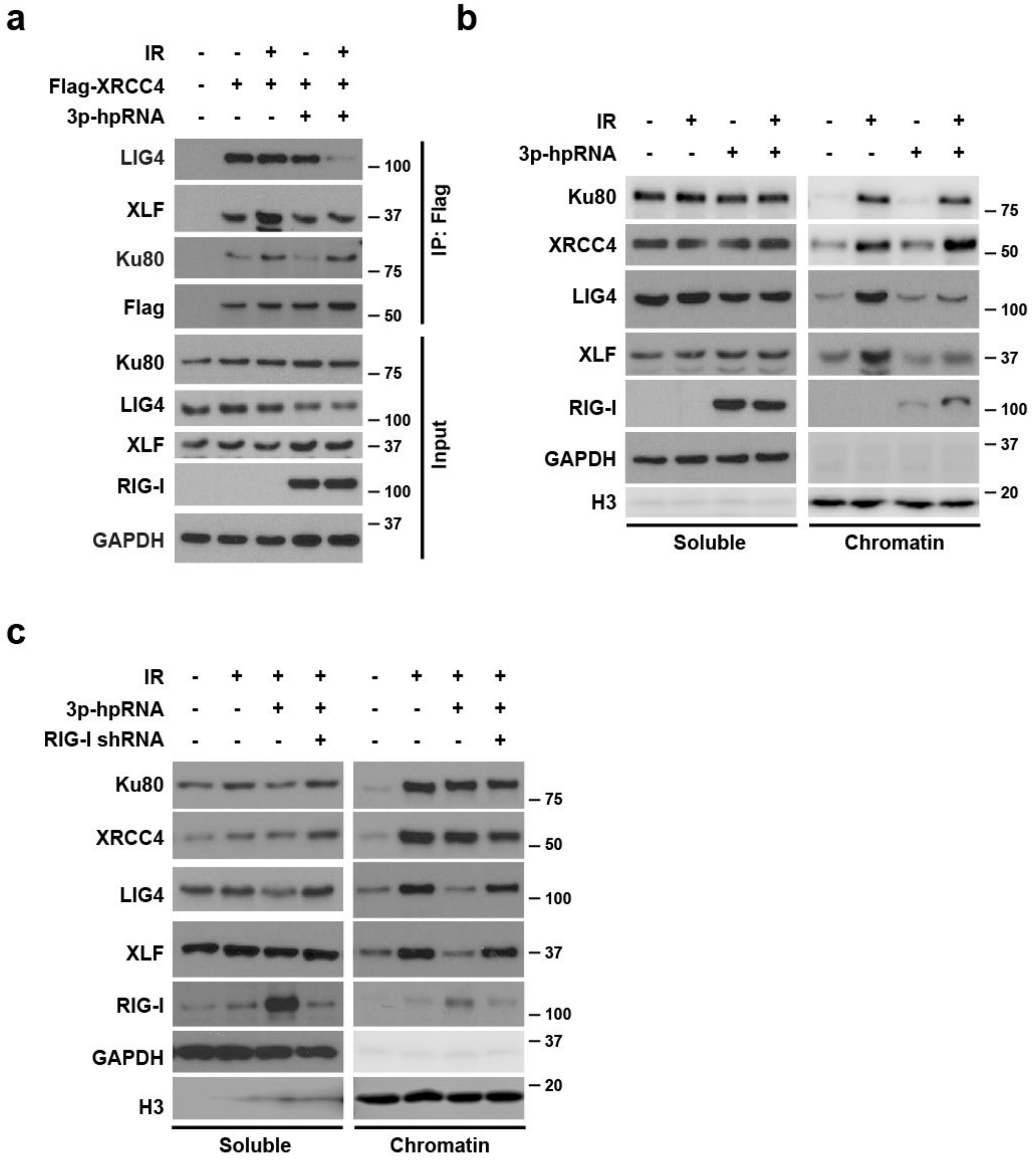

Supplementary Fig. 3: RIG-I suppresses non-homologous end joining by disrupting the formation of XRCC4/LIG4/XLF complex at DSB sites. **a** HEK293T cells were transfected with Flag-XRCC4 and 3p-hpRNA (0.5 μg/ml), and then treated with IR (10 Gy, 1-2 h). The cells were lysed and immunoprecipitated with anti-Flag agarose beads. The beads were boiled, subjected to SDS-PAGE, and analyzed with indicated antibodies. **b** A549 cells were transfected with 3p-hpRNA (0.5 μg/ml, 12 h), and then treated with IR (10 Gy, 1 h). The soluble and chromatin fractions were subjected to SDS-PAGE and analyzed with indicated antibodies. **c** A549 cells were transfected with 3p-hpRNA (0.5 μg/ml, 12 h), then infected with lentiviruses expressing RIG-I shRNA, and treated with IR (10 Gy, 1 h). The soluble and chromatin fractions were subjected to SDS-PAGE and analyzed with indicated antibodies.

Supplementary Figure 4

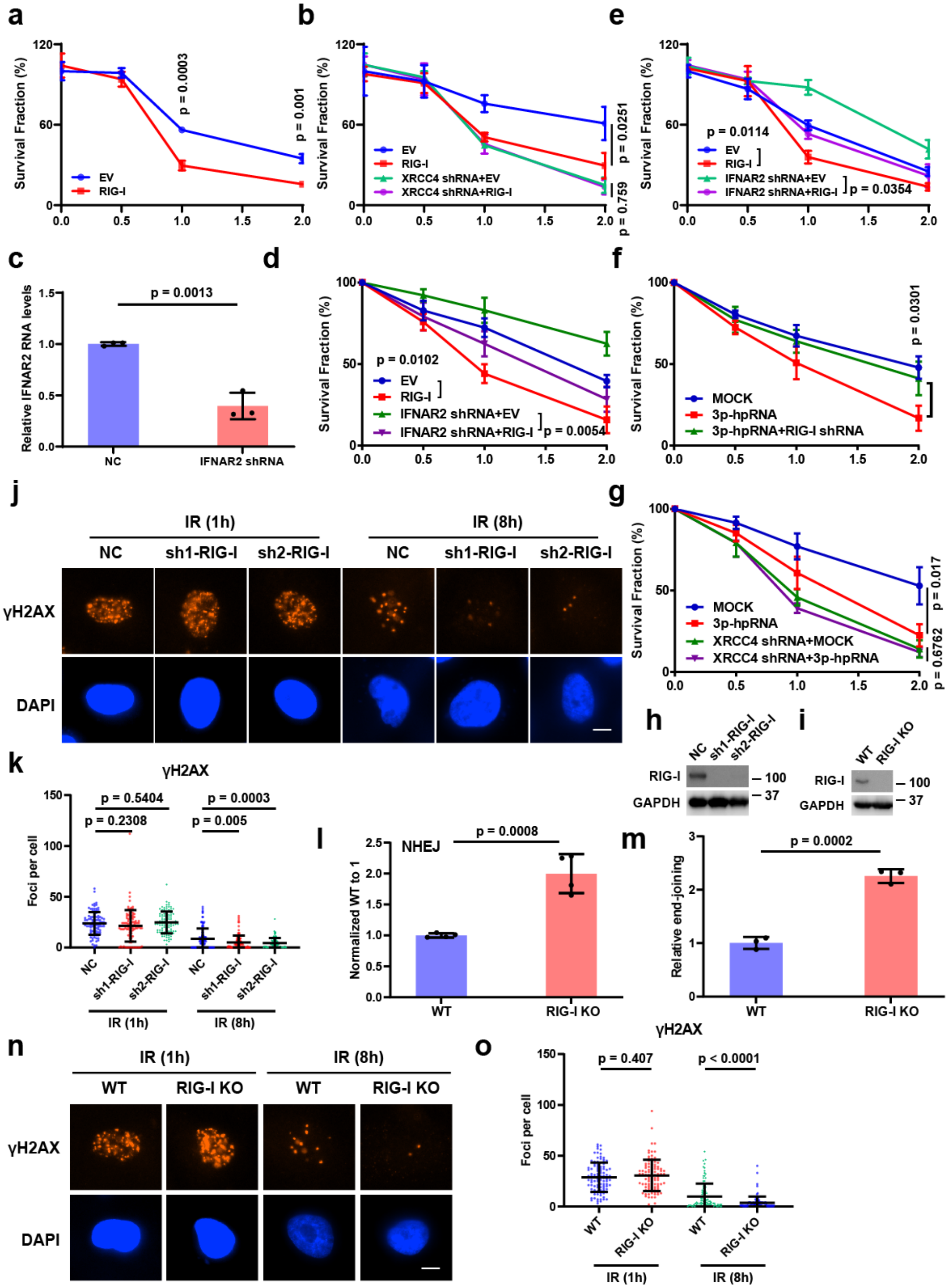

Supplementary Figure 4

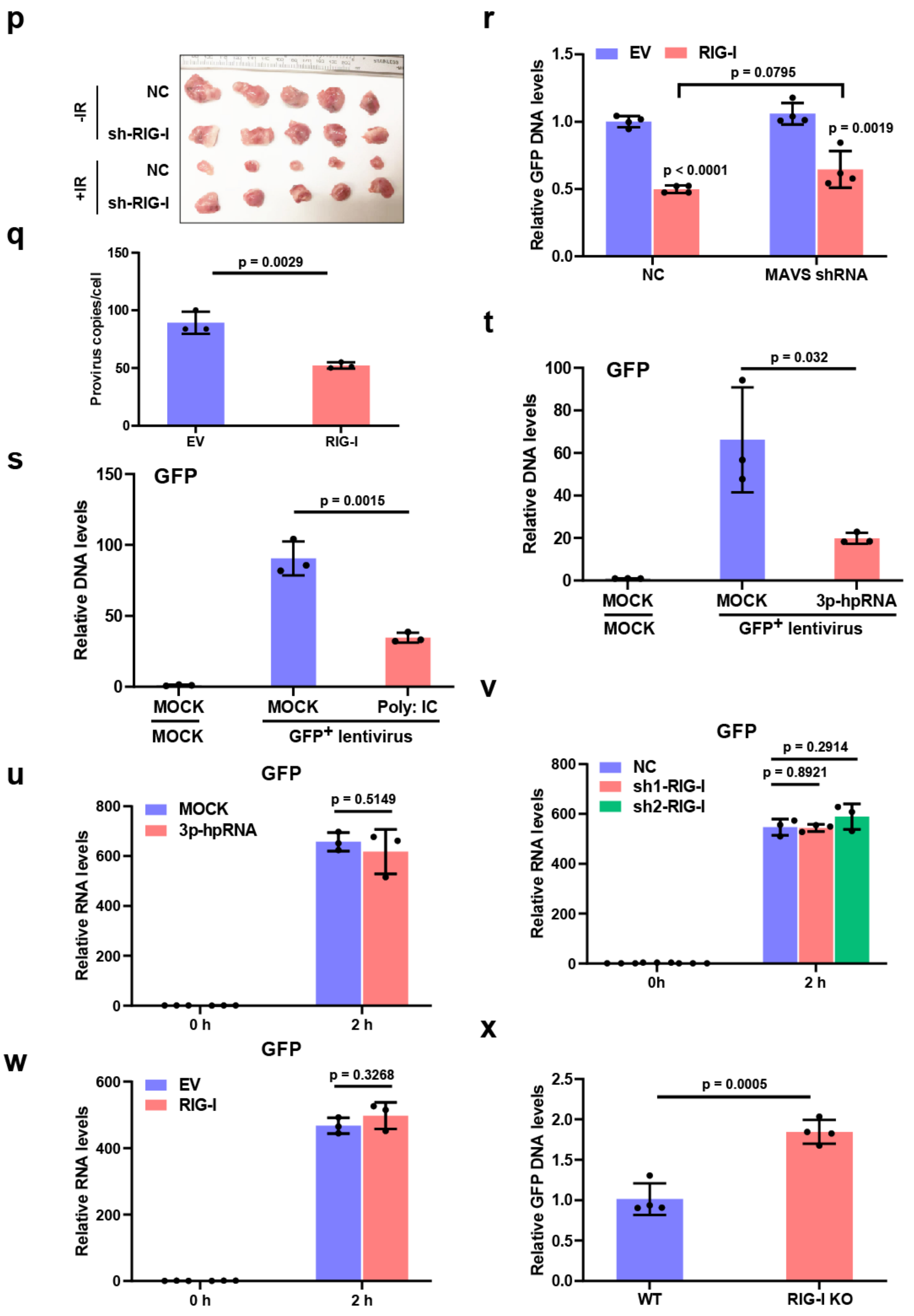

Supplementary Fig. 4: RIG-I suppresses retrovirus integration into the host genome by impeding non-homologous end joining. a Colony formation assay for control and RIG-I overexpressing A549 cells exposed to different dosage of IR. Data shown are representative of three independent experiments and values are mean ± SEM of technical replicates ($n = 3$). *P*-values are determined by unpaired two-sided t-test. **b** Control and XRCC4 knockdown A549 cells overexpressing RIG-I, were treated with indicated dosage of IR. Cell viability was assessed using colony formation assay. Data shown are representative of three independent experiments and values are mean ± SEM of technical replicates ($n = 3$). *P*-values are determined by unpaired two-sided t-test. **c** IFNAR2 RNA levels in control and IFNAR2 knockdown A549 cells were analyzed by qRT-PCR. Data are presented as mean values ± SEM from three independent experiments. *P*-values are determined by unpaired two-sided t-test. **d-e** The sensitivity of control and IFNAR2 knockdown A549 cells overexpressing RIG-I treated with IR, was analyzed by colony formation assay. Data shown are representative of three independent experiments and values are mean ± SEM of technical replicates ($n = 3$). *P*-values are determined by unpaired two-sided t-test. **f** A549 cells were transfected with 3p-hpRNA, then infected with lentiviruses expressing RIG-I shRNA, and treated with indicated dosage of IR. Cell viability was assessed using colony formation assay. Data shown are representative of three independent experiments and values are mean ± SEM of technical replicates ($n = 3$). *P*-values are determined by unpaired two-sided t-test. **g** The IR sensitivity of control and XRCC4 knockdown A549 cells transfected with 3p-hpRNA, was analyzed by colony formation assay. Data shown are representative of three independent experiments and values are mean ± SEM of technical replicates ($n = 3$). *P*-values are determined by unpaired two-sided t-test. **h-i** RIG-I protein levels in A549 cells expressing RIG-I shRNA **(h)** or sgRNA **(i)**, were detected by immunoblotting. **j-k** Representative pictures **(j)** and quantification **(k)** of γH2AX foci in control and RIG-I knockdown A549 cells treated with IR (2 Gy). Data are representative of three independent experiments. Each dot represents a single cell, and 100 cells were counted in each group for this experiment. Error bars represent ± SEM from this experiment. *P*-values are determined by unpaired two-sided t-test. Scale bar, 10 μm. **l** WT and RIG-I knockout HEK293T cells, were transfected with NHEJ reporter, and then cells were harvested for NHEJ assay. Data are presented as mean values ± SEM from three independent experiments. *P*-values are determined by unpaired two-sided t-test. **m** WT and RIG-I knockout HEK293T cells were transfected with linearized-pEYFP plasmid for 12 h, followed by qPCR to detect the ligated EYFP region, normalized to an uncut flanking DNA sequence. Data are presented as mean values ± SEM from three independent experiments. *P*-values are determined by unpaired two-sided t-test. **n-o** Representative pictures **(n)** and quantification **(o)** of γH2AX foci in WT and RIG-I knockout A549 cells treated with IR (2 Gy). Data are representative of three independent experiments. Each dot represents a single cell, and 100 cells were counted in each group for this experiment. Error bars represent ± SEM from this experiment. *P*-values are determined by unpaired two-sided t-test. Scale bar, 10 μm. **p** Control or RIG-I knockdown A549 cells were subcutaneously injected into the flank of NOD-SCID mice. Mice were treated with or without IR. Shown are tumor images from different groups. **q** Control and RIG-I overexpressing U2OS cells, were infected with GFP-positive lentiviruses. Provirus copies in the genomic DNA were analyzed by qPCR. Data are presented as mean values ± SEM from three independent experiments. *P*-values are determined by unpaired two-sided t-test. **r** Control and MAVS knockdown HEK293T cells overexpressing RIG-I, were infected with GFP positive lentiviruses. GFP levels in the genomic DNA were analyzed by qPCR. Data are presented as mean values ± SEM from four independent

experiments. *P*-values are determined by unpaired two-sided t-test. **s-t** HEK293T cells were transfected with Poly: IC (0.5 µg/ml, 12 h) **(s)** or 3p-hpRNA (0.5 µg/ml, 12 h) **(t)**, and then infected with GFP-positive lentiviruses. GFP levels in the genomic DNA were analyzed by qPCR. Data are presented as mean values ± SEM from three independent experiments. *P*-values are determined by unpaired two-sided t-test. **u-w** 3p-hpRNA treated **(u)**, RIG-I knockdown **(v)**, or RIG-I overexpressing **(w)** HEK293T cells, were infected with GFP positive lentiviruses for 2 h. GFP RNA levels were detected by qRT-PCR. Data are presented as mean values ± SEM from three independent experiments. *P*-values are determined by unpaired two-sided t-test. **x** WT and RIG-I knockout HEK293T were infected with GFP positive lentiviruses. GFP levels in the genomic DNA were analyzed by qPCR. Data are presented as mean values ± SEM from four independent experiments. *P*-values are determined by unpaired two-sided t-test.

Supplementary Figure 5

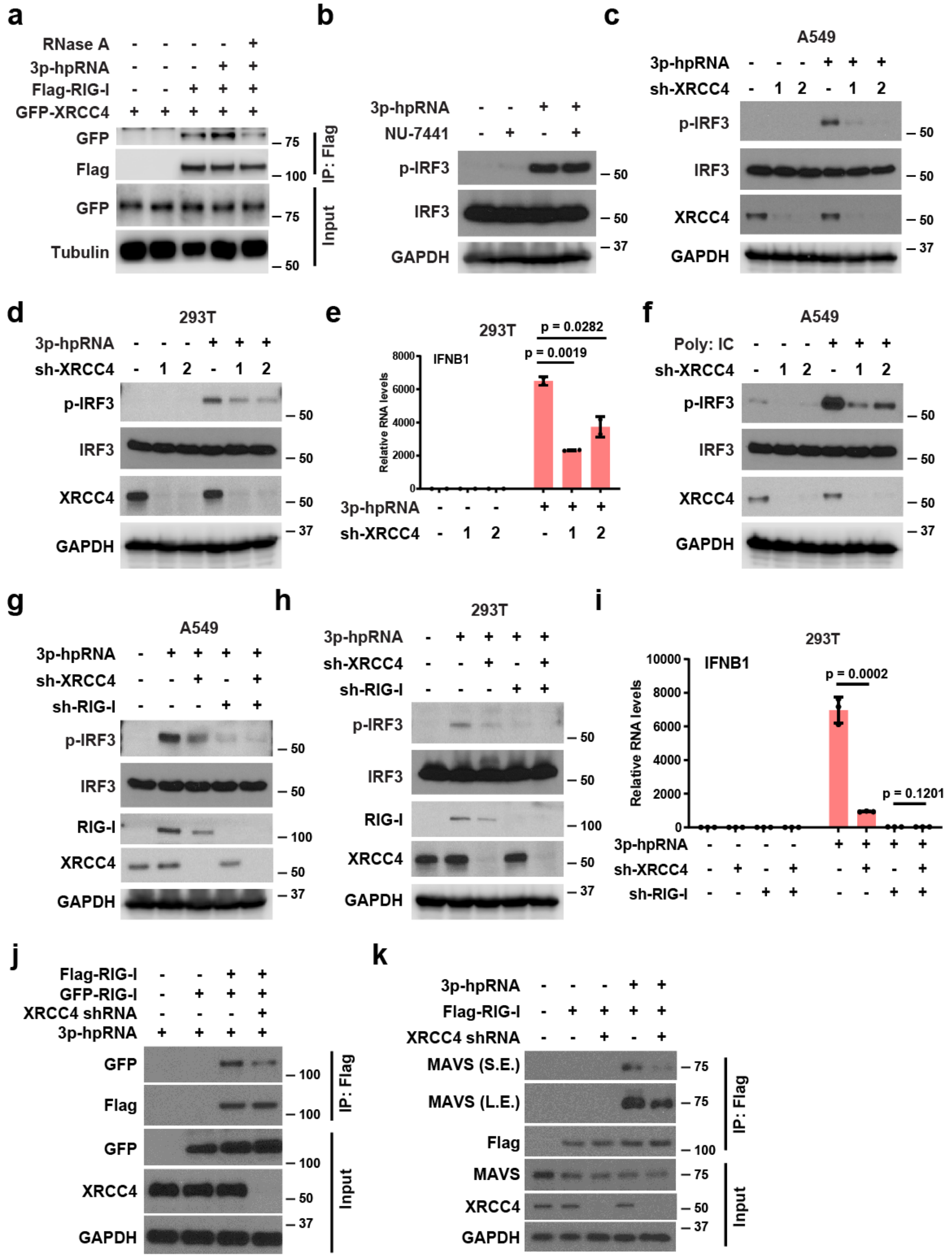

Supplementary Fig. 5: Loss of XRCC4 attenuates RIG-I immune signaling. **a** HEK293T cells were transfected with Flag-RIG-I and GFP-XRCC4, and then 3p-hpRNA (0.5 µg/ml, 8 h). Cells were lysed, and cytosolic fractions were immunoprecipitated with anti-Flag agarose beads.

The beads were treated with RNase A, boiled and blotted with indicated antibodies. **b** A549 cells were treated with DNA-PK inhibitor (NU-7441, 2 uM, 24 h) and then transfected with 3p-hpRNA (0.5 μg/ml, 8 h). The cell lysates were subjected to SDS-PAGE and blotted with indicated antibodies. **c** Control and XRCC4 knockdown A549 cells were transfected with 3p-hpRNA (0.5 μg/ml, 8 h). The cell lysates were subjected to SDS-PAGE and blotted with indicated antibodies. **d-e** Control and XRCC4 knockdown HEK293T cells were transfected with 3p-hpRNA. The IRF3 phosphorylation **(d)** and IFN-β RNA levels **(e)** were detected by Western blot and qRT-PCR respectively. Data **(e)** are presented as mean values ± SEM from two independent experiments. *P*-values are determined by unpaired two-sided t-test. **f** Control and XRCC4 knockdown A549 cells were transfected with Poly: IC (0.5 μg/ml, 8 h). The cell lysates were subjected to SDS-PAGE and blotted with indicated antibodies. **g** Control and XRCC4 knockdown A549 cells stably expressing RIG-I shRNA, were transfected with 3p-hpRNA (0.5 μg/ml, 12 h). The cell lysates were subjected to SDS-PAGE and blotted with indicated antibodies. **h-i** Control and XRCC4 knockdown HEK293T cells stably expressing RIG-I shRNA, were transfected with 3p-hpRNA. The IRF3 phosphorylation **(h)** and IFN-β RNA levels **(i)** were detected by Western blot and qRT-PCR respectively. Data are presented as mean values ± SEM from three independent experiments. *P*-values are determined by unpaired two-sided t-test. **j** Control and XRCC4 knockdown cells were transfected with Flag- and GFP-tagged RIG-I, and then 3p-hpRNA (0.5 μg/ml, 8 h). The cells were lysed and immunoprecipitated with anti-Flag agarose beads. The beads were boiled and blotted with indicated antibodies. **k** Control and XRCC4 knockdown cells were transfected with Flag-RIG-I and then 3p-hpRNA (0.5 μg/ml, 8 h). The cells were lysed and immunoprecipitated with anti-Flag agarose beads. The beads were boiled and blotted with indicated antibodies.

Supplementary Figure 6

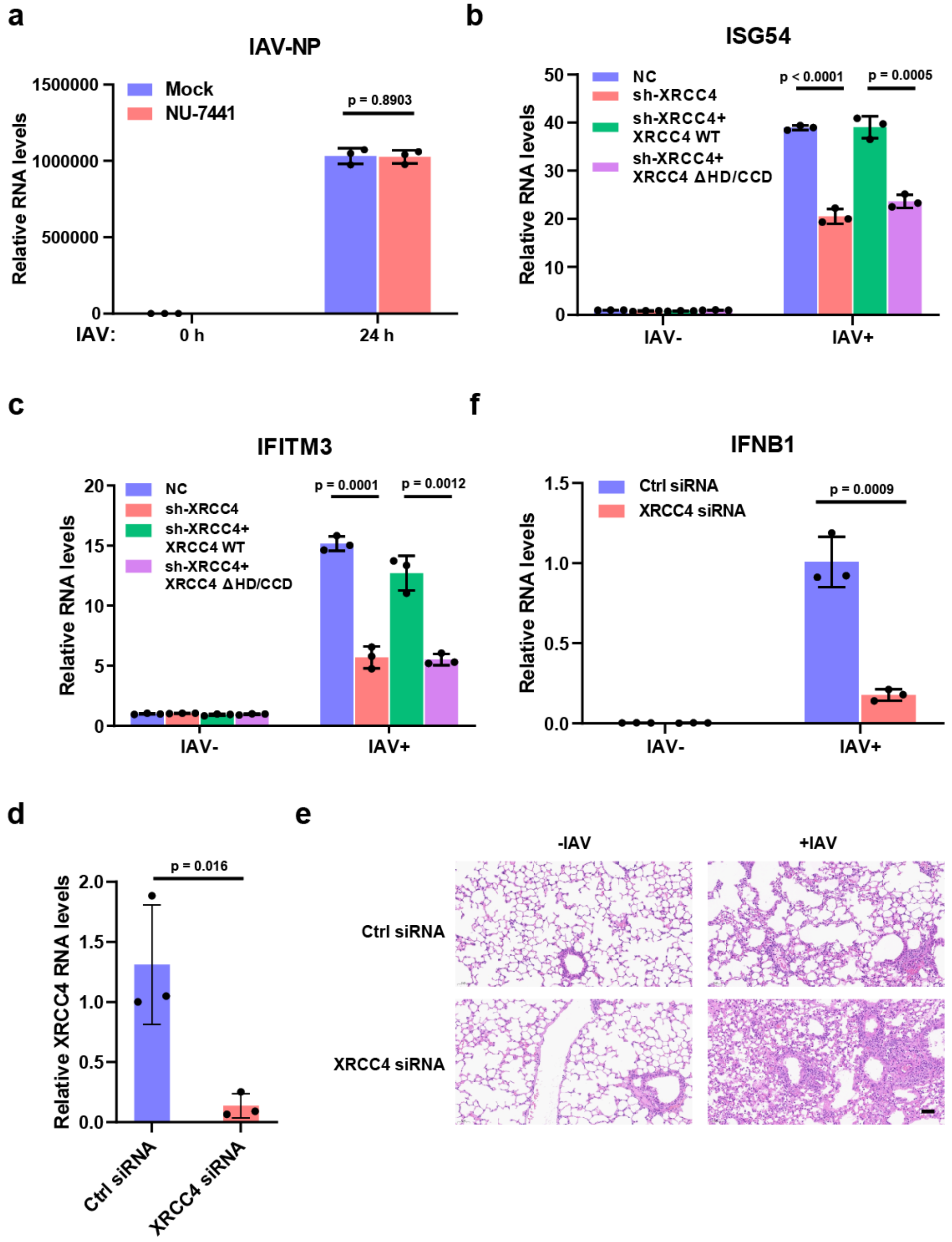

Supplementary Fig. 6: XRCC4 coordinates with RIG-I to suppress RNA virus replication in host cells. a A549 cells were treated with DNA-PK inhibitor (NU-7441, 2 uM, 24 h) and then infected with influenza virus A/PR/8/34 (IAV-PR8) as indicated. IAV NP RNA levels were detected by qRT-PCR. Data are presented as mean values ± SEM from three independent

experiments. *P*-values are determined by unpaired two-sided t-test. **b-c** ISG54 **(b)** and IFITM3 **(c)** RNA levels in XRCC4 knockdown cells re-expressing WT or XRCC4 mutant (ΔHD/CCD) infected with IAV-PR8, were detected by qRT-PCR. Data are presented as mean values ± SEM from three independent experiments. *P*-values are determined by unpaired two-sided t-test. **d** XRCC4 RNA levels in the lung tissues of mice treated with control or XRCC4 siRNA, were detected by qRT-PCR. Data are presented as mean values ± SEM from three independent experiments. *P*-values are determined by unpaired two-sided t-test. **e** C57BL/6 mice were treated with control or XRCC4 siRNA followed by infection with influenza virus A/WSN/1933 (IAV-WSN). Shown are representative pictures of lung tissues stained with hematoxylin and eosin (HE) with or without WSN infection. Scale bar, 50 μm. **f** IFN-β RNA levels in the lung tissues of mice treated with control or XRCC4 siRNA followed by infection with IAV WSN, were detected by qRT-PCR. Data are presented as mean values ± SEM from three independent experiments. *P*-values are determined by unpaired two-sided t-test.

Supplementary Figure 7

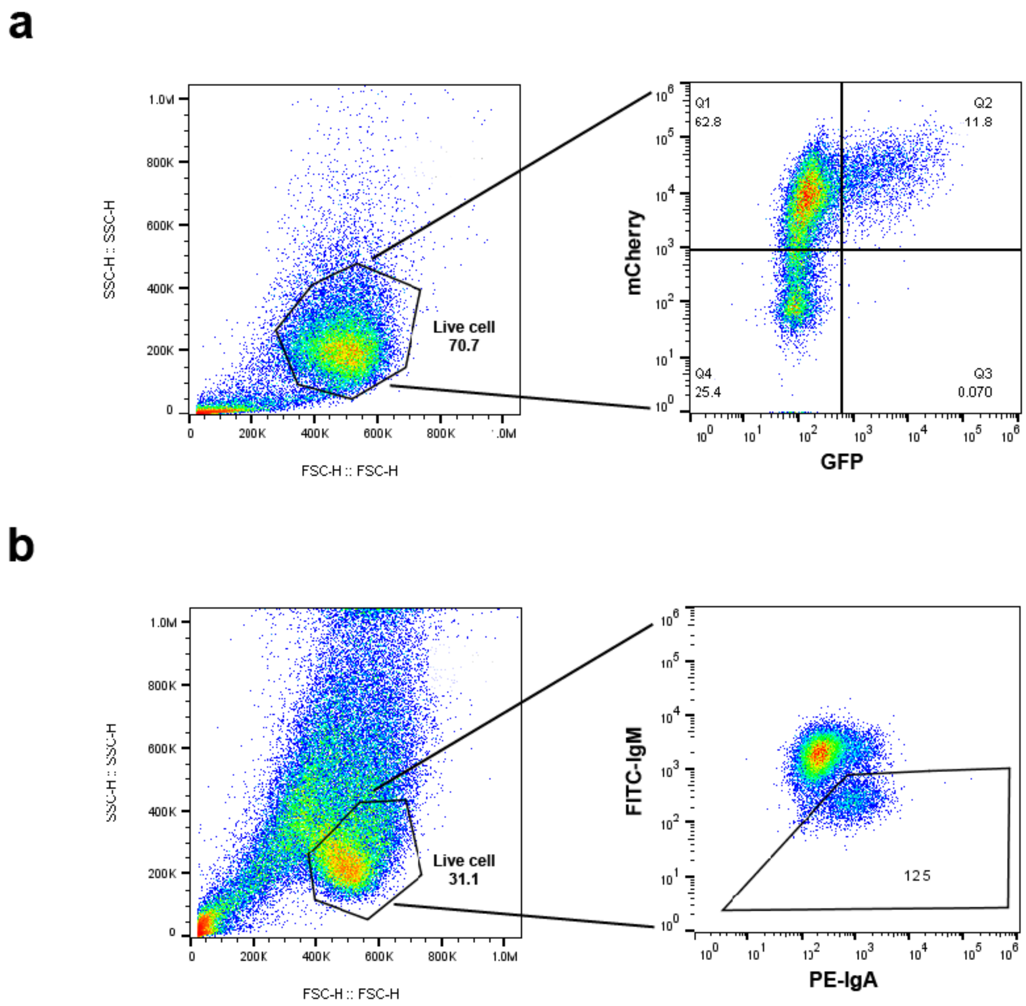

Supplementary Fig. 7: FACS gating strategy. a Gating strategy for HR and NHEJ assay. HR/NHEJ efficiency = Q2/ (Q1+Q2) (right panel). **b** Gating strategy for CSR assay. Numbers indicate the percentage of IgA+ cells (right panel).

Supplementary Table 1 Sequence of qPCR primers used in this study

NP	Primer FW	CATGTCAAAGGAAGGCACGA
	Primer REV	GAACAACCGTTATGGCAGCA
human IFNB1	Primer FW	GCTTGGATTCCTACAAAGAAGCA
	Primer REV	ATAGATGGTCAATGCGGCGTC
human IFITM3	Primer FW	CGTGAAGTCTAGGGACAGGA
	Primer REV	CCATAGGCCTGGAAGATCAGC
human ISG54	Primer FW	GAGCAGCCTACGGCAACTAA
	Primer REV	GCCTCGTTTTGCCCTTTGAG
GFP	Primer FW	CGACTTCTTCAAGTCCGCCA
	Primer REV	TTGTACTCCAGCTTGTGCCC
human GAPDH	Primer FW	CAGCCTCAAGATCATCAGCA
	Primer REV	TGTGGTCATGAGTCCTTCCA
mouse IFNB1	Primer FW	TACACTGCCTTTGCCATCCA
	Primer REV	GAGGACATCTCCCACGTCAA
mouse XRCC4	Primer FW	AGCCTGGACTGCAACAGTTT
	Primer REV	GGGAGAAATGCCGAGACTCC
mouse GAPDH	Primer FW	TGTGAACGGATTTGGCCGTA
	Primer REV	ACTGTGCCGTTGAATTTGCC
CHIP primers	DSB1_FW	GATTGGCTATGGGTGTGGAC
	DSB1_REV	CATCCTTGCAAACCAGTCCT
	DSB2_FW	TTCCTGCAGCCTCATTTTCT
	DSB2_REV	TGATGATGCCTTTTCCCTTC
NHEJ linear assay primers	YFP FW	GCTGGTTTAGTGAACCGTCAG
	YFP REV	GCTGAACTTGTGGCCGTTTA
	internal control FW	TACATCAATGGGCGTGGATA
	internal control REV	AAGTCCCGTTGATTTTGGTG
