# Supplementary material for: Reciprocal regulation of RIG-I and XRCC4 connects DNA repair with RIG-I immune signaling
Source: Nat Commun. 2021 Apr 12;12:2187. doi: 10.1038/s41467-021-22484-7 (PMC8041803; doi:10.1038/s41467-021-22484-7)
Supplement: Supplementary file 2 — Reporting Summary [file 41467_2021_22484_MOESM2_ESM.pdf]

nature research

Corresponding author(s): Zhenkun Lou

Last updated by author(s): Mar 1, 2021

Reporting Summary

Nature Research wishes to improve the reproducibility of the work that we publish. This form provides structure for consistency and transparency in reporting. For further information on Nature Research policies, see our Editorial Policies and the Editorial Policy Checklist.

Statistics

For all statistical analyses, confirm that the following items are present in the figure legend, table legend, main text, or Methods section.

n/a	Confirmed	
☐	☒	The exact sample size (n) for each experimental group/condition, given as a discrete number and unit of measurement
☐	☒	A statement on whether measurements were taken from distinct samples or whether the same sample was measured repeatedly
☐	☒	The statistical test(s) used AND whether they are one- or two-sided<br>*Only common tests should be described solely by name; describe more complex techniques in the Methods section.*
☐	☒	A description of all covariates tested
☐	☒	A description of any assumptions or corrections, such as tests of normality and adjustment for multiple comparisons
☐	☒	A full description of the statistical parameters including central tendency (e.g. means) or other basic estimates (e.g. regression coefficient) AND variation (e.g. standard deviation) or associated estimates of uncertainty (e.g. confidence intervals)
☐	☒	For null hypothesis testing, the test statistic (e.g. F, t, r) with confidence intervals, effect sizes, degrees of freedom and P value noted<br>*Give P values as exact values whenever suitable.*
☒	☐	For Bayesian analysis, information on the choice of priors and Markov chain Monte Carlo settings
☒	☐	For hierarchical and complex designs, identification of the appropriate level for tests and full reporting of outcomes
☒	☐	Estimates of effect sizes (e.g. Cohen's d, Pearson's r), indicating how they were calculated

Our web collection on statistics for biologists contains articles on many of the points above.

Software and code

Policy information about availability of computer code

Data collection	Attune NxT v2.6
Data analysis	GraphPad Prism 8, Microsoft Excel 2010, Flowjo V10, Image J

For manuscripts utilizing custom algorithms or software that are central to the research but not yet described in published literature, software must be made available to editors and reviewers. We strongly encourage code deposition in a community repository (e.g. GitHub). See the Nature Research guidelines for submitting code & software for further information.

Data

Policy information about availability of data

All manuscripts must include a data availability statement. This statement should provide the following information, where applicable:

- Accession codes, unique identifiers, or web links for publicly available datasets
- A list of figures that have associated raw data
- A description of any restrictions on data availability

All the data are available from the corresponding authors upon reasonable request. Source data for figures are provided with this paper.

April 2020

Field-specific reporting

Please select the one below that is the best fit for your research. If you are not sure, read the appropriate sections before making your selection.

☒ Life sciences ☐ Behavioural & social sciences ☐ Ecological, evolutionary & environmental sciences

For a reference copy of the document with all sections, see nature.com/documents/nr-reporting-summary-flat.pdf

Life sciences study design

All studies must disclose on these points even when the disclosure is negative.

Sample size	No statistical method was used to predetermine sample size.
Data exclusions	No data were excluded from analysis.
Replication	All results were tested and confirmed with at least three independent experiments.
Randomization	All samples such as cells or animals were randomly allocated into experimental groups.
Blinding	For manual microscopy, data were collected randomly choose at least 5 regions. For high-content microscopy, data were analyzed automatically. One individual performed the experiment while another individual (blinded to the group allocation) performed the analysis. For colony-forming units quantification, data were collected blindly by technicians without information of the research purpose.<br>For immunoblotting, immunoprecipitation and qPCR experiments, samples of different groups were fairly collected and generated simultaneously, and all data are obtained and presented in an unbiased manner.

Reporting for specific materials, systems and methods

We require information from authors about some types of materials, experimental systems and methods used in many studies. Here, indicate whether each material, system or method listed is relevant to your study. If you are not sure if a list item applies to your research, read the appropriate section before selecting a response.

Materials & experimental systems

n/a	Involved in the study	
☐	☒	Antibodies
☐	☒	Eukaryotic cell lines
☒	☐	Palaeontology and archaeology
☐	☒	Animals and other organisms
☒	☐	Human research participants
☒	☐	Clinical data
☒	☐	Dual use research of concern

Methods

n/a	Involved in the study	
☒	☐	ChIP-seq
☐	☒	Flow cytometry
☒	☐	MRI-based neuroimaging

Antibodies

Antibodies used	The following antibodies were used:<br>Anti-RIG-I, 1:1000 (CST: 3743, rabbit)<br>Anti-IRF3 (Ser396), 1:1000 (CST: 4947, rabbit)<br>Anti-MAVS, 1:1000 (CST: 3993, rabbit)<br>Anti-XLF, 1:1000 (Abcam: ab33499, rabbit)<br>Anti-Ub, 1:2000 (Santa cruz Biotechnology: sc-8017, mouse)<br>Anti-XRCC4, 1:1000 (Santa cruz Biotechnology: sc-271087, mouse)<br>Anti-FLAG, 1:2000 (Sigma: F1804, mouse)<br>Anti-GFP, 1:1000 (Santa cruz Biotechnology: sc-9996, mouse)<br>Anti-DNA-PK, 1:2000 (Thermo Fisher: MA5-13238, mouse)<br>Anti-KU80, 1:2000 (Thermo Fisher: MA5-12933, mouse)<br>Anti-Lamin B1, 1:1000 (Proteintech: 12987-1-AP, rabbit)<br>Anti-GAPDH, 1:2000 (Proteintech: 60004-1-lg, mouse)<br>Anti-LIG4, 1:500 (Proteintech: 12695-1-AP, rabbit)<br>Anti-RIG-I, 1:1000 (Abcam: ab45428, rabbit)<br>Anti-XRCC4, 1:1000 (Proteintech: 15817-1-AP, rabbit)<br>Alexa Fluor® 488 AffiniPure Donkey Anti-Rabbit IgG (H+L 715-585-150), Alexa Fluor® 594 AffiniPure Donkey Anti-Rabbit IgG (H+L 711-585-152), Alexa Fluor® 488 AffiniPure Donkey Anti-Mouse IgG (H+L 715-545-150), and Alexa Fluor® 594 AffiniPure Donkey Anti-Mouse IgG (H+L 715-585-150) were purchased from Jackson Lab.<br>Donkey Anti-Mouse IgG (H+L) ML (715-675-151) and Donkey Anti-Rabbit IgG (H+L) ML (711-675-152) were purchased from Jackson ImmunoResearch.

Validation	The detailed information is listed:<br>Anti-RIG-I, 1:1000 (CST: 3743, rabbit). Reacts with: Human, Mouse, Rat, Monkey. Suitable for: WB, IP. Citation from manufacturer are listed at https://www.cellsignal.com/products/primary-antibodies/rig-i-d14g6-rabbit-mab/3743<br>Anti-IRF3 (Ser396), 1:1000 (CST: 4947, rabbit). Reacts with: Human, Mouse. Suitable for: WB. Citation from manufacturer are listed at https://www.cellsignal.com/products/primary-antibodies/phospho-irf-3-ser396-4d4g-rabbit-mab/4947<br>Anti-MAVS, 1:1000 (CST: 3993, rabbit). Reacts with: Human. Suitable for: WB, IF. Citation from manufacturer are listed at https://www.cellsignal.com/products/primary-antibodies/mavs-antibody/3993<br>Anti-XLF, 1:1000 (Abcam: ab33499, rabbit). Reacts with: Human. Suitable for: WB, IP, IF. Citation from manufacturer are listed at https://www.abcam.com/xlf-antibody-ab33499.html<br>Anti-Ub, 1:2000 (Santa cruz Biotechnology: sc-8017, mouse), is recommended for detection of Ubiquitin, poly-ubiquitinated and ubiquitinated proteins of mouse, rat, human and Drosophila origin by WB, IP, IF, IHC(P), FCM and ELISA. Citation from manufacturer are listed at https: https://www.scbt.com/p/ubiquitin-antibody-p4d1<br>Anti-XRCC4, 1:1000 (Santa cruz Biotechnology: sc-271087, mouse), is recommended for detection of XRCC4 of mouse, rat and human origin by WB, IP, IF, IHC(P) and ELISA. Citation from manufacturer are listed at https://www.scbt.com/p/xrcc4-antibody-c-4<br>Anti-FLAG, 1:2000 (Sigma: F1804, mouse), highly sensitive and specific detection of FLAG fusion proteins by immunoblotting, immunoprecipitation (IP), immunohistochemisty, immunofluorescence and immunocyotchemistry. Citation from manufacturer are listed at https://www.sigmaaldrich.com/catalog/product/sigma/f1804?lang=en&region=US<br>Anti-GFP, 1:1000 (Santa cruz Biotechnology: sc-9996, mouse), is recommended for detection of GFP and GFP mutant fusion proteins by WB, IP, IF, FCM and ELISA. Citation from manufacturer are listed at https://www.scbt.com/p/gfp-antibody-b-2?requestFrom=search<br>Anti-DNA-PK, 1:2000 (Thermo Fisher: MA5-13238, mouse), targets DNA-PKcs in IF, IHC (P), IP, and WB applications and shows reactivity with Human and Rat samples. Citation from manufacturer are listed at https://www.thermofisher.com/antibody/product/DNA-PK-Antibody-clone-18-2-Monoclonal/MA5-13238<br>Anti-KU80, 1:2000 (Thermo Fisher: MA5-12933, mouse), targets Ku (p80) in FACS, ICC/IF, IP, and WB applications and shows reactivity with Human, Mouse and Non-human primate samples. Citation from manufacturer are listed at https://www.thermofisher.com/antibody/product/Ku80-Antibody-clone-111-Monoclonal/MA5-12933<br>Anti-Lamin B1, 1:1000 (Proteintech: 12987-1-AP, rabbit), targets Lamin B1 in WB, IP, IHC, IF, FC, CHIP, ELISA applications and shows reactivity with human, mouse, rat samples. Citation from manufacturer are listed at https://www.ptglab.com/products/LMNB1-Antibody-12987-1-AP.htm<br>Anti-GAPDH, 1:2000 (Proteintech: 60004-1-lg, mouse). Reactivity: Human, Mouse, Rat, Yeast, Plant. Applications: WB, IP, IHC, IF, FC, ELISA. Citation from manufacturer are listed at https://www.ptglab.com/products/GAPDH-Antibody-60004-1-Ig.htm<br>Anti-LIG4, 1:500 (Proteintech: 12695-1-AP, rabbit), targets LIG4 in WB, IHC, IF, ELISA applications and shows reactivity with human, mouse, rat samples. Citation from manufacturer are listed at https://www.ptglab.com/products/LIG4-Antibody-12695-1-AP.htm<br>Anti-RIG-I, 1:1000 (Abcam: ab45428, rabbit), Reacts with: Mouse, Rat, Human. Suitable for: WB, IHC, IF. Citation from manufacturer are listed at https://www.abcam.com/rig-iddx58-antibody-ab45428.html<br>Anti-XRCC4, 1:1000 (Proteintech: 15817-1-AP, rabbit), targets XRCC4 in WB, IP, IHC, ELISA applications and shows reactivity with human, rat samples. Citation from manufacturer are listed at https://www.ptglab.com/products/XRCC4-Antibody-15817-1-AP.htm<br>The commercial secondary antibodies were validated by manufactures.

Eukaryotic cell lines

Policy information about cell lines

Cell line source(s)	HEK293T (ATCC)<br>A549 (ATCC)<br>U2OS (ATCC)<br>U2OS ER-AsiSI cells [generated by Dr Gaëlle Legube (Université de Toulouse, Toulouse, France)]<br>U2OS FokI cells [provided by Roger A. Greenberg (Department of Pathology, University of Pennsylvania, Philadelphia, PA)]
Authentication	The cell lines have been authenticated based on morphological criteria.
Mycoplasma contamination	All cell lines were tested negative for mycoplasma using PCR assays with species-specific primers.
Commonly misidentified lines<br>(See ICLAC register)	No commonly misidentified cell lines were used.

Animals and other organisms

Policy information about studies involving animals; ARRIVE guidelines recommended for reporting animal research

Laboratory animals	Female 6-8 weeks old outbred athymic nude mice, and female 5-6 weeks old C57BL/6 mice are used in experiments
Wild animals	No wild animals were used in this study
Field-collected samples	No filed-collected samples were used in this study
Ethics oversight	All animal work was approved by the Institutional Animal Care and Use Committee (IACUC) at Mayo Clinic

Note that full information on the approval of the study protocol must also be provided in the manuscript.

Flow Cytometry

Plots

Confirm that:

☒ The axis labels state the marker and fluorochrome used (e.g. CD4-FITC).

☒ The axis scales are clearly visible. Include numbers along axes only for bottom left plot of group (a 'group' is an analysis of identical markers).

☒ All plots are contour plots with outliers or pseudocolor plots.

☒ A numerical value for number of cells or percentage (with statistics) is provided.

Methodology

Sample preparation	For HR/NHEJ analysis, cells were transfected with mCherry/GFP plasmids, then collected and suspended in PBS. For CSR analysis, cells were stimulated with ligands, then collected, and stained with murine IgA and IgM antibody. Further details of experimental procedures are provided in the Methods section.
Instrument	Attune NxT Flow Cytometer (Thermo Fisher)
Software	Attune NxT Flow Cytometer software v2.6
Cell population abundance	No flow based sorting was performed.
Gating strategy	The FSC/SSC gates defined the single cell population and gated indicated antibodies positive population.

☒ Tick this box to confirm that a figure exemplifying the gating strategy is provided in the Supplementary Information.
